# Dorsal Hand Vein Image Enhancement Using Fusion of CLAHE and Fuzzy Adaptive Gamma

**DOI:** 10.3390/s21196445

**Published:** 2021-09-27

**Authors:** Marlina Yakno, Junita Mohamad-Saleh, Mohd Zamri Ibrahim

**Affiliations:** 1 Faculty of Electrical and Electronic Engineering Technology, Universiti Malaysia Pahang, Pekan 26600, Pahang, Malaysia; marlinayakno@ump.edu.my (M.Y.); zamri@ump.edu.my (M.Z.I.); 2 School of Electrical and Electronic Engineering, Universiti Sains Malaysia, Nibong Tebal 14300, Pulau Pinang, Malaysia

**Keywords:** image enhancement, image fusion, near-infrared image, vein detection

## Abstract

Enhancement of captured hand vein images is essential for a number of purposes, such as accurate biometric identification and ease of medical intravenous access. This paper presents an improved hand vein image enhancement technique based on weighted average fusion of contrast limited adaptive histogram equalization (CLAHE) and fuzzy adaptive gamma (FAG). The proposed technique is applied using three stages. Firstly, grey level intensities with CLAHE are locally applied to image pixels for contrast enhancement. Secondly, the grey level intensities are then globally transformed into membership planes and modified with FAG operator for the same purposes. Finally, the resultant images from CLAHE and FAG are fused using improved weighted averaging methods for clearer vein patterns. Then, matched filter with first-order derivative Gaussian (MF-FODG) is employed to segment vein patterns. The proposed technique was tested on self-acquired dorsal hand vein images as well as images from the SUAS databases. The performance of the proposed technique is compared with various other image enhancement techniques based on mean square error (MSE), peak signal-to-noise ratio (PSNR), and structural similarity index measurement (SSIM). The proposed enhancement technique’s impact on the segmentation process has also been evaluated using sensitivity, accuracy, and dice coefficient. The experimental results show that the proposed enhancement technique can significantly enhance the hand vein patterns and improve the detection of dorsal hand veins.

## 1. Introduction

Dorsal hand veins form an extensive network of blood vessels under the skin behind a person’s palm. Uniqueness, universality, and permanence are significant properties of dorsal hand veins [1]. Thus, vein patterns enable verification of individuals’ unique identities based on the images of their biometric characteristics and properties [2]. In biomedicine, dorsal hand vein images have been used to aid paramedics in inserting a catheter into a vein in an intravenous procedure to deliver medical fluids. As such, works to improve the accuracy of vein detection and location have become an increasingly vital issue.

The problem with dorsal hand veins is that they may be hardly seen in visible light due to under-skin fat and blood occlusion. Only under near-infrared (NIR) light veins can be visible through an image due to different infrared light responses towards different skin layers. Using NIR, veins appear as dark pixels, while the other regions appear as brighter pixels. Hence, the NIR concept has been adopted in research works for vein imaging [3,4,5]. Although the identification and verification based on hand veins through NIR imaging have been studied for many years, improvements are still necessary particularly to overcome the challenging effects of environmental lighting, ambient temperature, and light scattering. The effects have the tendency to downgrade the quality of an acquired vein image resulting in an image with low contrast that vein patterns are not easily distinguishable from other tissues. As a result, the accuracy of vein detection and recognition also reduces. In addition, a transformation of low contrast vein images directly to a binary image usually has partially led to loss of some vein pattern information.

Many previous studies have developed different enhancement methods for vein images to overcome the performance degradation of hand vein detection and pattern recognition [6,7,8,9,10]. In general, these studies can be broadly categorized into standard image enhancement and fusion image enhancement.

The rest of the sections in this paper are organized as follows. Section 2 discusses previous related works. This section includes introduction to the standard vein enhancement, image fusion, and some introduction to the proposed technique. Section 3 introduces the proposed technique in detail, including the hand vein databases and the performance measures. Finally, the experimental results and some concluding remarks are given in Section 4 and Section 5, respectively.

## 2. Related Works

### 2.1. Vein Image Enhancement

Enhancement of vein image involves improving the contrast of vein patterns from its background before conversion to binary image through segmentation. Vein image enhancement can generally be divided into three domains: spatial domain, frequency domain, and fuzzy domain. The spatial domain method operates directly on pixels, the frequency domain method operates directly on the transformation coefficient, and the fuzzy domain method operates based on a fuzzy set containing a mathematical tool for handling vagueness. Out of the three, the spatial domain is the simplest. It includes conventional methods such as contrast stretching [11,12], and gamma correction [6], which are widely used for vein contrast. However, these conventional methods usually transform each pixel of an image using a single transformation function. Moreover, the selection of the parameters requires experience, and if they are set manually, it may not give a suitable enhancement, especially if the images are dark or bright contrast images. Thus, the overall illumination cannot be perceived, and these may create over-enhancement or under-enhancement problems [13]. The authors of [14] proposed an adaptive gamma correction (AGC) method to enhance dark and bright contrast images to mitigate this problem. The classification of dark images and bright images is based on the mean intensity of the image. In [14] the authors assumed that pixels with mean intensity greater than or equal to 0.5 are classified as bright pixels, and the mean intensity of less than 0.5 is classified as dark pixels. Then, the classifications of image pixels are enhanced using different types of transformation functions. In addition, the gamma correction factor in [14] is calculated dynamically for each image according to its statistical information.

Although histogram-based enhancement techniques, namely histogram equalization (HE), is able to provide better vein enhancement than the conventional spatial domain techniques in [15], it poorly equalizes every local detail. This is because HE transformed the original histogram of the entire image into a uniformly distributed histogram, resulting in a loss of visibility. To overcome this limitation, the works of [16,17] have used adaptive histogram equalization (AHE); however, AHE over-amplify noise in the resultant image. Later, advancement on HE and AHE, referred to as CLAHE has produced outstanding results for contrast enhancement of dorsal hand vein images [18,19,20]. It restrains the problem of noise over-amplification in AHE using a clip limit criterion. Nevertheless, applying CLAHE alone in image enhancement will not remove noise completely because CLAHE works on small contextual regions instead of the entire image, which can be restricted to abstain from increasing any noise that may be available in the image.

Vein enhancement based on spatial domain filtering such as adaptive learning Gabor filters [7], 2D Gabor filters [21], and adaptive Gabor filters [22] has been successfully applied for vein images. The Gaussian directional filter [23], guided filter with single-scale Retinex [24], unsharp masking [11], and Gaussian matched filters [25] have also been used to boost up the local contrast in vein patterns. These filtering-based methods focus more on the edges so that the process may change the edges’ contrast. Therefore, it leads to degraded edges.

In the frequency domain, the work of [26] introduced a method for increasing the contrast of palm vein using two-dimensional discrete wavelet transform and linear discrimination discrete. Later, the authors of [27] applied one-scale wavelet transform and HE for enhancing and sharpening dorsal vein images. Another work [8] introduced a method for increasing the contrast of finger vein images using symmetric mask-based discrete wavelet transform and adaptive contrast technique. Overall, the frequency domain methods by [8,26,27] are able to detect small fluctuations, which proves them as a suitable approach for vein contrast enhancement. However, their executions are time-consuming due to complex implementation.

As already known, image characteristics such as brightness, darkness, and boundary, are fuzzy in nature. Hence, the fuzzy domain is a good alternative, especially to the conventional approaches [9]. Fuzzy domain is able to improve the brightness of both local and global-based images as it can brighten the surrounding by having uniform density. Researchers of work [9] proposed fuzzy histogram hyperbolization (FHH) in blood vessels in the retinal image and have succeeded in improving the contrast. In another work, the authors of [15] employed a sequence of CLAHE and FHH to improve dorsal hand vein images. Although FHH is able to provide better vein or vessel enhancement, it highly depends on the selected membership function and the value of the hedge factor, β. When a selection of β reaching 0, it will produce bright images, and when β reaching ∞, it will produce dark images. On the other hand, FHH also has the common problem with conventional gamma correction, which requires experience for selecting the hedge factor, β. Sometimes, it will end up with over-enhancement or under-enhancement problems.

### 2.2. Image Fusion

As already known, an enhanced image may lose certain important information depending on the employed enhancement method. In other words, an enhancement method may stress different information from another method; hence, image fusion can be used to gather various images enhanced images [28]. In general, image fusion can be categorized into pixel-level fusion, feature-level fusion, and decision-level fusion. According to [29], a majority of reported image fusion methods are directed towards pixel-level fusion, revolving manipulation of pixels in input images to obtain the final fused result. The Averaging, Maximum Pixel Value, Minimum Pixel Value, Min-Max, and Simple Block Replace are the simplest algorithms in pixel-level fusion methods [10]. However, these techniques reduce the resultant image quality because the fusion process has generated unwanted noise into fused images [10]. It leads to unwanted side effects and directly affects the contrast of the image. A more comprehensive pixel-fusion approach is Weighted Averaging. It is a technique in which different weights values are assigned to all pixels in a source image. Then, a fused image is obtained by computing the weighted sum of all corresponding pixels in an input image. This method has been reported to be able to improve detection reliability [10].

## 3. Materials and Methods

As mentioned earlier, NIR image acquisitions usually suffer from low contrast. Enhancing NIR images, especially for hand images consisting of complex vein structures using a single enhancement algorithm from a single domain, may not be sufficient to bring out the hidden details. To mitigate these problems, the CLAHE algorithm is used as the first technique to enhance the contrast of hand vein images. CLAHE is selected particularly due to a reported result that CLAHE has shown to be superior at enhancing local contrast by reducing the effects of edge shading in both noisy and homogenous areas, especially on medical images [19]. Hence, it is expected to be able to improve the local contrast and details of the vein patterns in this work. Second, a global technique of fuzzy adaptive gamma (FAG) was proposed. Obviously, the primary target of this work is to improve the global contrast for diverse hand vein images, specifically bright and dark, using different transformation functions. As AGC has been reported to be superior at enhancing global details [14], AGC in a fuzzy domain named FAG is selected to contribute to the fusion method in this work by improving the overall visualization of dorsal hand vein patterns. Third, a fusion approach using weighted average is proposed by taking advantage of the first technique (i.e., CLAHE) to achieve clear local details and the second technique (i.e., FAG) to gain high global visibility. Finally, a modification of weighted average fusion is proposed to further improve the contrast of hand vein images.

**Figure 1:**
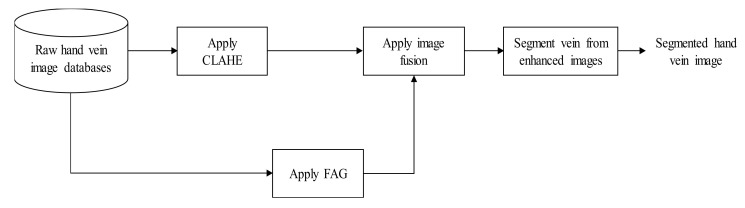
Block diagram of the proposed fusion of CLAHE and FAG for hand vein image enhancement.

The block diagram for hand vein contrast enhancement based on a fusion of CLAHE and FAG is illustrated in Figure 1. CLAHE and FAG are two preprocessing algorithms to enhance the hand vein image in spatial and fuzzy domains. Output from both preprocessing stages is combined using fusion improved weighted average. Then, an enhanced hand vein image is obtained. Finally, vein segmentation is conducted using MF-FDOG to segment veins from the background.

### 3.1. Dorsal Hand Vein Image Databases

In this work, two databases of dorsal hand vein images were used. The first database consists of images obtained from self-acquisition and a database from Sakarya University of Applied Science (SUAS). The self-acquisition system comprises four parts: three sets of 850 nm near-infrared LED arrays, platform base as a target area, raspberry pi 3B+ (RPI), and RPI night vision camera. The Kodak 87C Wratten filter is installed in front of the night vision camera lens to prevent visible light from reaching the camera [30,31]. The near-infrared LEDs that are mounted to the left, right, and front of the night camera are used to light up the target area. The night camera and LEDs are connected to the Raspberry Pi board via flex cable. A total of 100 volunteers aged between 7 and 60 years old with an equal number of males and females participated as subjects for dorsal hand vein image acquisition. Six grayscale image samples were captured from each volunteer, and the images were stored in a JPG format with a resolution of 640 × 480.

The second database consists of the SUAS dorsal hand vein images obtained from the Department of Electrical and Electronic Engineering, Sakarya University of Applied Sciences, Turkey. It consists of 919 images taken from 155 adults (80 males and 75 females) who participated in the study [19]. Volunteers were asked to place their right and left hands at fist position under an infrared camera on a white surface for 3 s. The resolution of each image is 640 × 480 in JPG format. All images from both datasets were then auto-cropped and resized to 192 × 192 for standardization.

### 3.2. Hand Vein Enhancement

#### 3.2.1. Contrast Limited Adaptive Histogram Equalization (CLAHE)

Contrast limited adaptive histogram equalization (CLAHE) algorithm was used as the first technique for enhancing the contrast between veins and the background. The detailed step of the CLAHE algorithms are explained as follows [32]:

- Image is decomposed into a number of continuous and non-overlapping contextual regions.
- A histogram for each of these contextual regions is computed.
- Clip limit is a threshold parameter for effectively altering the contrast of the image. Thus, an appropriate value of clip limit is determined based on trial-and-error to increase the local image regions.
- The histogram of each contextual region is clipped above the threshold.
- The transformation function for each clipped histogram is applied to perform equalize greyscale mapping. The mathematical expression for transform intensity value into uniform distribution can be given as [33]:
  (1) $$g=\left[g_{max}-g_{min}\right]*P\left(f\right)+g_{min}$$
  where $g_{max}$ is maximum pixel value, $g_{max}$ is minimum pixel value, g is computed pixel value, $P\left(f\right)\;$ is a cumulative probability distribution.
- The neighboring regions are combined using bilinear interpolation to create an enhanced image without artificially induced boundaries.

**Figure 2:**
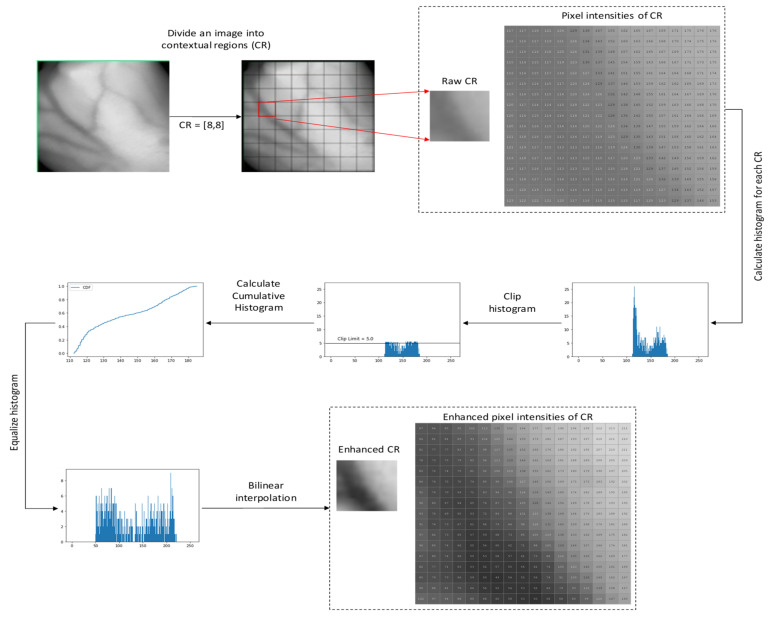
The flow of CLAHE procedures applied on a hand image.

Figure 2 illustrates the flow of CLAHE procedures applied on a hand vein image.

#### 3.2.2. Proposed Fuzzy Adaptive Gamma (FAG)

The fuzzy adaptive gamma (FAG) algorithm was used as the second technique to enhance the contrast between veins and the background. The proposed FAG image enhancement involves three stages: fuzzification, modification of membership values, and defuzzification.

**Figure 3:**
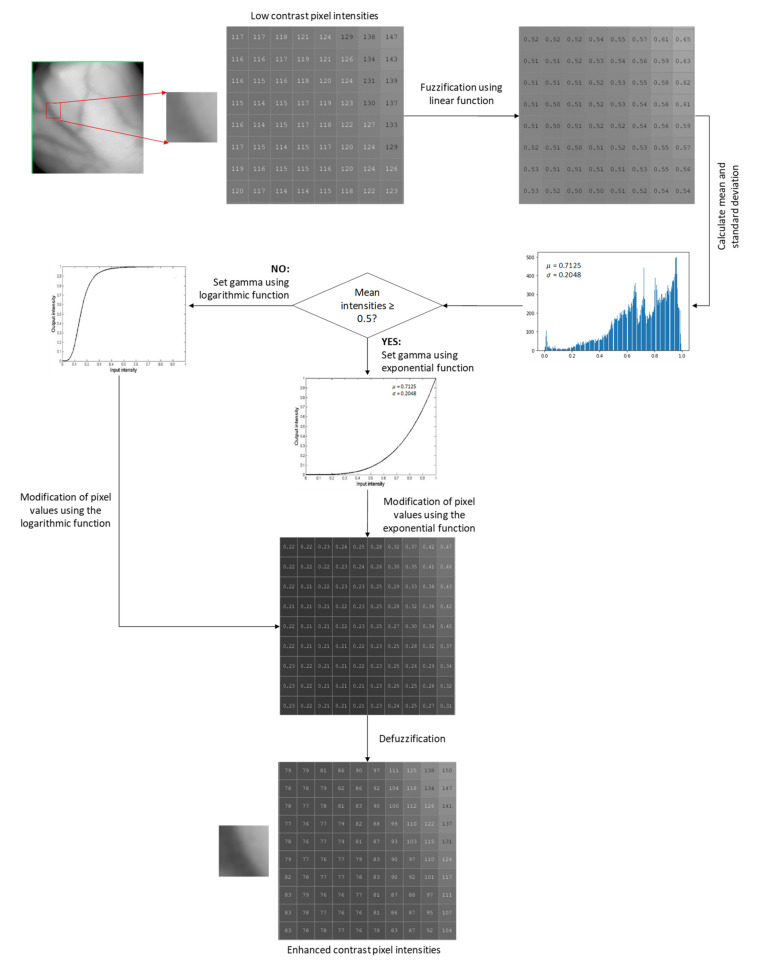
The procedures for the proposed FAG on a small part of vein pixels.

- Fuzzification: Fuzzification is a step to determine the degree to which input data belongs to each of the appropriate fuzzy sets via the membership function. Hence, this work involves converting greyscale values of dorsal hand vein image as an input to the fuzzy domain whose value ranges between 0 and 1 using a linear function. The linear function can be expressed as [15]
  (2) $$I_{out}^{}=\frac{I_{in}^{}-I_{min}}{I_{max}-I_{min}}$$
  where $I_{in}^{}$ is the image of the dorsal hand vein,$\;I_{out}^{}$ is the dorsal hand vein image in the fuzzy domain, $I_{min}$ is the minimum value of the gray-level and $I_{max}$ is the maximum value of the gray level in the image.
- Modification of the membership values: Low contrast of hand vein images can either be bright or dark intensities. Depending on the brightness of the image, different image intensities should be modified differently. Therefore, the fuzzy modification is carried out to enhance image pixel intensity using AGC [14]. According to [14], the mean intensity µ, greater or equal to 0.5, is classified as an image with brighter pixels. Meanwhile, the mean intensity of less than 0.5 is classified as an image with darker pixels. Based on that theory, the transformation function of the AGC can be categorized as follows [14]:
  (3) $$I_{out}^{\gamma }=\left\{\begin{array}{cc} cI_{in}^{\gamma } & \;if\;\mu \geq 0.5\\ \frac{I_{in}^{\gamma }}{I_{in}^{\gamma }+\left(1-I_{in}^{\gamma }\right)\times \mu^{\gamma }}\; & if\;\mu <0.5\; \end{array}\right.$$
  where $I_{in}^{\gamma }$ and $I_{out}^{\gamma }$ are the input and output image intensities, respectively. c and γ are two parameters that control the shape of the transformation curve. The value of γ is calculated by [14]:
  (4) $$\gamma =-\log_{2}\left(\sigma \right)$$
  where σ is the standard deviation of the image intensity. The value of c is calculated by [14]:
  (5) $$c=\frac{1}{1+Heaviside\left(0.5-\mu \right)\times \left(k-1\right)}$$
  where k is defined by [14]:
  (6) $$k=I_{in}^{\gamma }+\left(1-I_{in}^{\gamma }\right)\times \mu^{\gamma }$$
  and the Heaviside function is given by [14]:
  (7) $$Heaviside\left(x\right)=\left\{\begin{array}{c} 0,\;x\leq 0\\ 1,\;x>0 \end{array}\right.$$
- Defuzzification: The new gray levels of defuzzification have been produced as follows [15]:
  (8) $$I_{out}^{}=\left[\frac{L-1}{e^{-1}-1}\right]\times \left[e^{-I_{out}^{\gamma }{}^{}}-1\right]$$
  where $I_{out}^{\gamma }\;$ is the gray level in the fuzzy membership values, $I_{out}^{}$ is the new gray level values, and L is the maximum number of gray levels in the original image. The procedures for the proposed FAG on a small part of vein pixels are illustrated in Figure 3.

#### 3.2.3. Proposed Improved Weighted Average Fusion

In this work, both resultant images from CLAHE and proposed FAG are fused using weighted averaging method. A modification was made on the weighted averaging equation as follows:

(9) $$f\left(x\right)=\left[\left(1-\omega_{0}\right)g_{0}\left(x\right)+\omega_{0}g_{1}\left(x\right)+\alpha \right]-\beta \left|g_{0}\left(x\right)-g_{1}\left(x\right)\right|$$

where $g_{0}\left(x\right)$ and $g_{1}\left(x\right)$ are the output images from CLAHE and proposed FAG, respectively. $\omega_{0}$ and $1-\omega_{0}$ are the corresponding weights for both output images to be verified in the range of [0,1]. α and β are constants. $\omega_{0,},\;\beta$ and α were set to optimal values of 0.5, 0.01, 7 (self-acquisition database), 1 (SUAS database) by trial-and-error investigation. These optimal values provided better enhancement results because more uniform intensities are distributed on the entire vein images.

**Figure 4:**
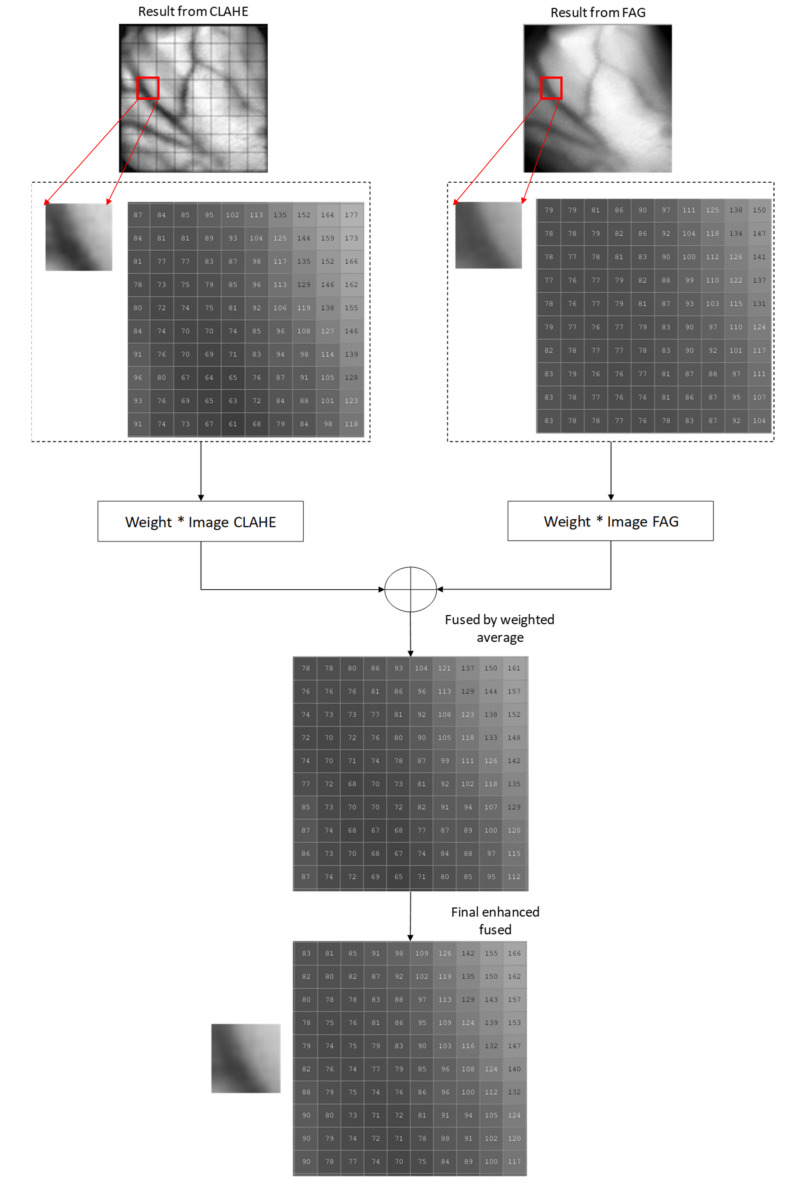
The proposed improved weighted average fusion flow diagram on a small part of vein pixels.

The first two terms of Equation (9), $\left[\left(1-\omega_{0}\right)g_{0}\left(x\right)+\omega_{0}g_{1}\left(x\right)+\alpha \right]$ are the weighted average of these two images, which affects the energy of the fused image while the last term, $\beta \left|g_{0}\left(x\right)-g_{1}\left(x\right)\right|$ is the weighted difference of these two images. The weighted difference is added to balance the weighted average. This improved weighted averaging fusion generated a fused function of CLAHE and proposed FAG. The flow diagram for the proposed improved weighted average fusion is illustrated in Figure 4.

### 3.3. Hand Vein Segmentation

The matched filter (MF) method has the advantages of simplicity and effectiveness to detect vessels by filtering and thresholding the original images. However, the matched filter with first-order derivative of Gaussian (MF-FODG) method was more accurate in distinguishing between the vein structures than MF alone. From the theory [34], by considering that the cross-section of a vessel is asymmetric Gaussian function, an actual vein will have a strong response to the MF around its peak position, while the local mean of its response is the FDOG that will be close to zero around the peak position. In contrast, both response signals of MF and the FDOG are high for non-vein structures. Such a difference implies that the vein and non-vein edges can be either distinguished by using the MF-FODG. Therefore, this work adopts MF-FODG to detect hand vein patterns. Then, morphological processing was used to refine the output of MF-FDOG. Details for MF-FODG are described in [34].

### 3.4. Performance Measures

To the best of our knowledge, several dorsal hand vein databases are available publicly, but none of them provide the ground truth images. Manually annotated, especially for hand vein structure, is very challenging and time-consuming. Therefore, only 40 self-acquisition and SUAS databases images were semi-automatically labeled with ‘1’ for vein pixel and ‘0’ for background pixel using the Isodata technique for analysis. Then, each pixel value and vein continuity of the original image and the generated were manually inspected for confirmation.

#### 3.4.1. Enhancement Image Analysis

The performance of the proposed FAG and improved weighted average fusion rules was evaluated by estimating quality metric parameters such as mean square error (MSE), peak signal-to-noise ratio (PSNR), and structural similarity index measurement (SSIM). MSE measures the error of the enhanced image as compared to the original image. Lower values of MSE indicate a lower error in the enhanced image as compared to the original. PSNR is a metric that measures the quality achievement of an enhanced image in comparison with original images as well as it indicates the degree to which contrast in the image is achieved. A higher value of PSNR indicates there is minimal degradation of the image compared to the original image. SSIM index is used to measure the structural similarity between enhanced image and original image. This metric combines local image structure, luminance, and contrast. It enables to determine how well the structures are preserved between the enhanced image and its original image. The larger SSIM means the enhanced image is closer to the original image. MSE, PSNR, and SSIM computation are given by the following equations [35]:

(10) $$\mathrm{MSE}=\frac{\sum_{n=1}^{N}\left(x^{n}-y^{n}\right)}{N}$$

(11) $$\mathrm{PSNR}=10\times \log_{10}\left(\frac{255^{2}}{MSE}\right)$$

(12) $$\mathrm{SSIM}=\frac{\left(2\mu_{x}\mu_{y}+c_{1}\right)\left(2\sigma_{xy}+c_{2}\right)}{\left(\mu_{x}^{2}+\mu_{y}^{2}+c_{1}\right)\left(\sigma_{x}^{2}+\sigma_{y}^{2}+c_{2}\right)}$$

where N is the size of the hand vein image, $x^{n}$ and $y^{n}$ are the *nth* pixel of original image x, processed image y; $\mu_{x}$, $\mu_{y}$ are the mean brightness of x, y; $\sigma_{x}^{2},\;\sigma_{y}^{2}$ are the global variance of x, y; $\sigma_{xy}$ is the covariance of x and y.

#### 3.4.2. Binary Image Analysis

In order to evaluate the overall performance of the proposed technique, the segmented images from various enhancement techniques were compared with the corresponding ground truth images. The results of each segmented pixel can be categorized into four different groups as follows:

- True positive (TP): vein pixel correctly segmented as vein pixel;
- False positive (FP): non-vein pixel segmented as vein pixel;
- True negative (TN): non-vein pixel correctly segmented as non-vein pixel;
- False negative (FN): vein pixel segmented as non-vein pixel.

Three different measurements were used for the overall evaluation of the segmentation performance. Sensitivity (Se), accuracy (Acc), and dice coefficient (Dice) were handpicked as the key performance matrices. The three performance matrices were calculated as follows [36]:

(13) $$\mathrm{Sensitivity}\;\left(\mathrm{Se}\right)=\frac{\mathrm{TP}}{\mathrm{TP}+\mathrm{FN}}$$

(14) $$\mathrm{Accuracy}\;\left(\mathrm{Acc}\right)=\frac{\mathrm{TP}+\mathrm{TN}}{\mathrm{TP}+\mathrm{TN}+\mathrm{FP}+\mathrm{FN}}$$

(15) $$\mathrm{Dice}\;\mathrm{coefficient}\;\left(\mathrm{Dice}\right)=\frac{2\mathrm{TP}}{2\mathrm{TP}+\mathrm{FP}+\mathrm{FN}}$$

Sensitivity quantifies a measure of how well a segmentation method can correctly identify vein pixels. Accuracy provides the overall performance of a segmentation method, while the dice coefficient evaluates the degree of similarity between the segmented result and the truth image. If the values of these performance metrics are high, it will indicate that the method is good.

## 4. Results and Discussion

This section first discusses the classification of hand vein images from self-acquisition and SUAS databases based on the image histograms observation and mean intensity. Next, we compared the enhancement performance of the proposed improved weighted average fusion of CLAHE + FAG with some other state-of-the-art techniques, namely CLAHE, FHH, and AGC. In addition, the overall performance of enhancement techniques in the segmentation stage was also evaluated. Enhancement analysis and binary analysis were performed in both qualitative and quantitative manners.

### 4.1. Experimental Datasets

**Figure 5:**
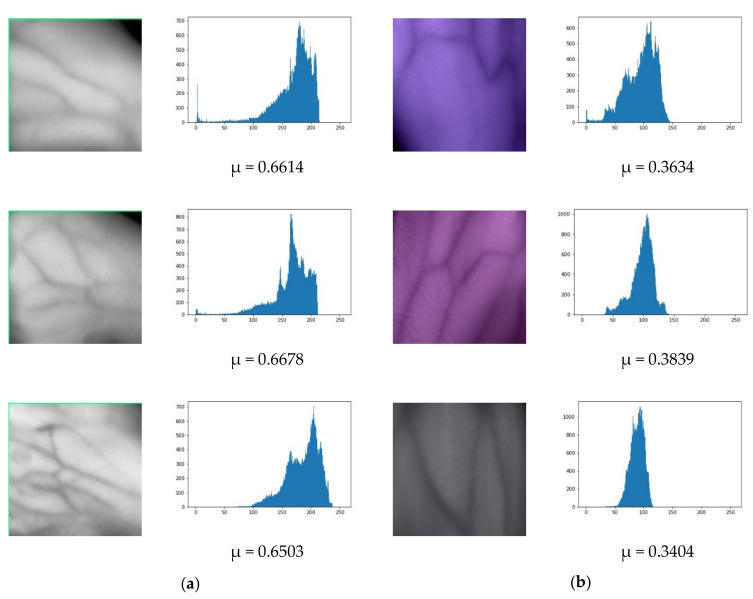
Original hand vein images with their corresponding histograms and mean intensities: (**a**) self-acquisition database, (**b**) SUAS database.

Figure 5a,b shows samples of hand vein images and their corresponding histograms from self-acquisition and SUAS databases. As can be seen, the hand vein images in Figure 5a are brighter than Figure 5b, and their corresponding histograms are accumulated within a minimal range and tilted towards the right side. With mean intensity greater than 0.5, the self-acquisition database’s hand vein images are considered as bright images. On the other hand, vein images from Figure 5b are darker, and their corresponding histograms are accumulated within a minimal range and are tilted towards the left side. With a mean intensity of less than 0.5, the SUAS database is considered as dark images.

### 4.2. Enhancement Analysis

**Figure 6:**
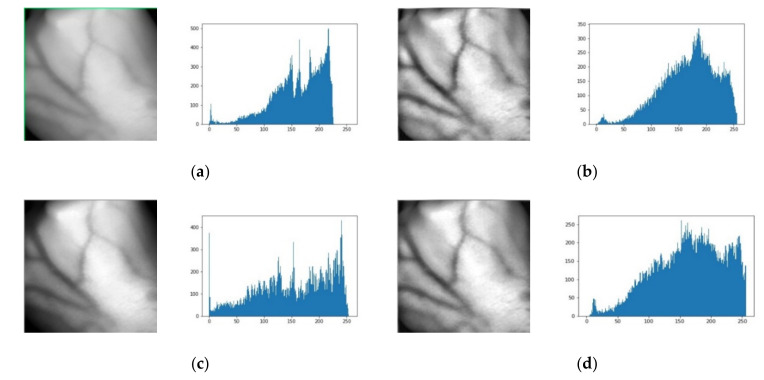
Results of each step of the proposed method with its corresponding histograms for a self-acquisition database. (**a**) Original bright contrast image. (**b**) After applying CLAHE. (**c**) After applying FAG. (**d**) CLAHE and FAG after applying improved weighted average fusion.

Figure 6 shows the results of each step involved in the proposed technique for the hand vein image self-acquisition database. Figure 6a shows the bright original hand vein image and its corresponding histogram. The equalized image after applying the CLAHE technique is shown in Figure 6b. This technique significantly enhances the original image; however, an over-enhancement was observed mainly in the background. Figure 6c is the equalized image after applying the FAG technique. This technique also substantially changes the original but causes the edges of vein patterns to be blurred. The equalized image after applying fusion of CLAHE and FAG using an improved weighted average is shown in Figure 6d. A final step shows that the proposed technique is more effective and produces high contrast of hand vein images.

**Figure 7:**
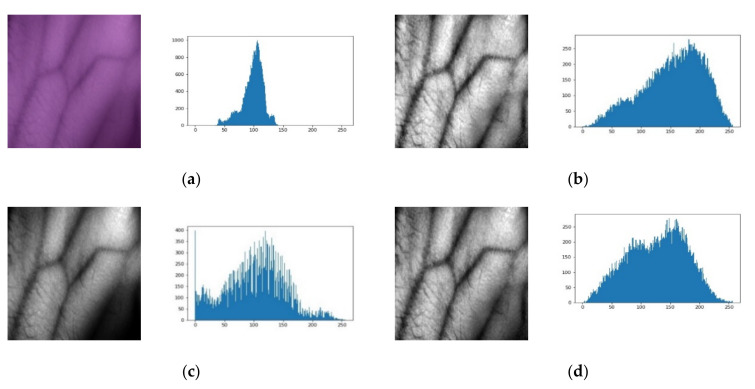
Result of each step of the proposed method with its corresponding histograms for the SUAS database. (**a**) Original dark contrast image. (**b**) After applying CLAHE. (**c**) After applying FAG. (**d**) CLAHE and FAG after applying improved weighted average fusion.

Figure 7 shows the results of each step involved in the proposed technique for the hand vein image SUAS database. The dark original hand vein image and its corresponding histogram are shown in Figure 7a. Figure 7b is the equalized image after applying the CLAHE technique. This technique significantly enhances the original image; however, an over-enhancement is also observed in the background. The equalized image after applying the FAG technique is shown in Figure 7c. This technique also substantially changes the original. Figure 7d shows the equalized image after applying fusion of CLAHE and FAG using an improved weighted average. A final step shows that the proposed technique is more effective and produces high contrast hand vein images. Information such as mean intensities and standard deviation from the image influence the transformation of FAG in the proposed image fusion.

**Figure 8:**
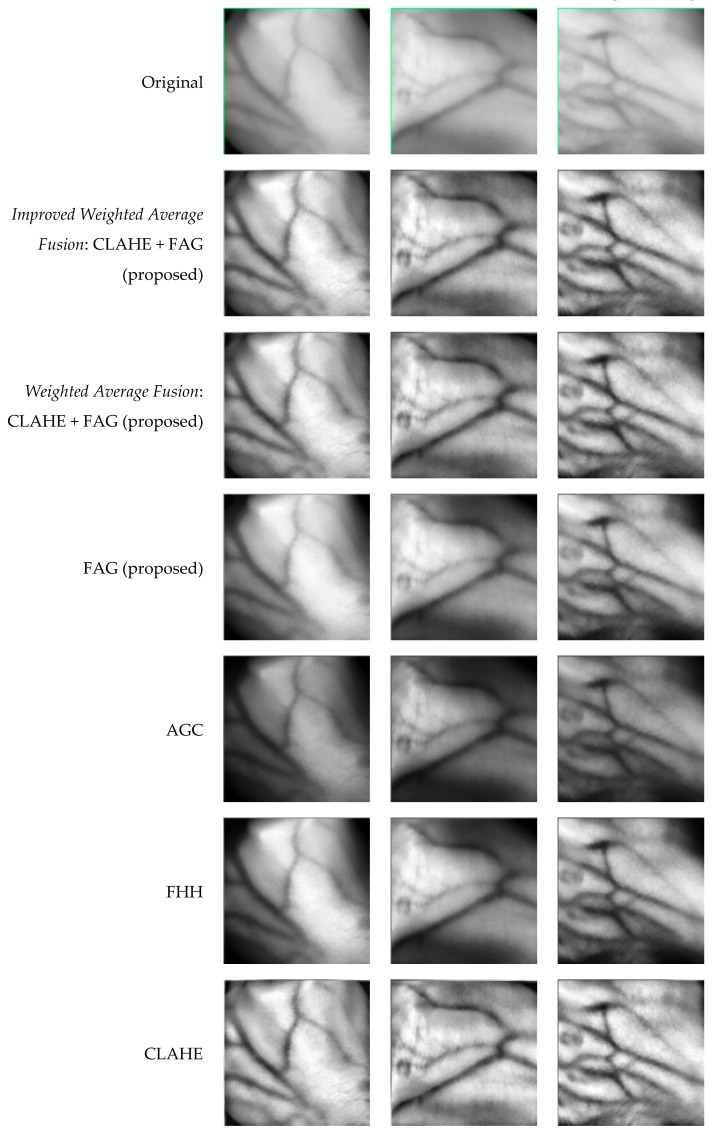
Sample results of various vein enhancement techniques based on three images from a self-acquisition database.

The proposed technique’s results compared to other enhancement techniques on a self-acquisition database are shown in Figure 8. It can be seen that CLAHE can enhance the local details better. However, the resulting image looks unnatural and has over-enhancement problems. Although AGC’s enhanced images have a brighter distribution than the original input images, AGC has caused level saturation effects in some small regions. This leads to the presence of some dark regions that are significantly darker than those in the original image. The same circumstance appears in the enhanced images using FHH. It can be seen that the images enhanced using FAG have better contrast compared to the AGC and FHH. However, FAG caused the edges of vein patterns to be blurred. The images enhanced using the proposed fusion of CLAHE + FAG with weighted average and improved weighted average have comparable results by visual observation. The fusion of CLAHE and FAG has better contrast in both local and global regions. It suppresses the over-enhancement problem in CLAHE, the level saturation problem in AGC, FHH, and the blurring vein edge problem in FAG while preserving the brightness. The enhanced images also have much clearer edges, particularly in vague local regions. Furthermore, the thickness of the veins remains the same as those in the original image.

**Figure 9:**
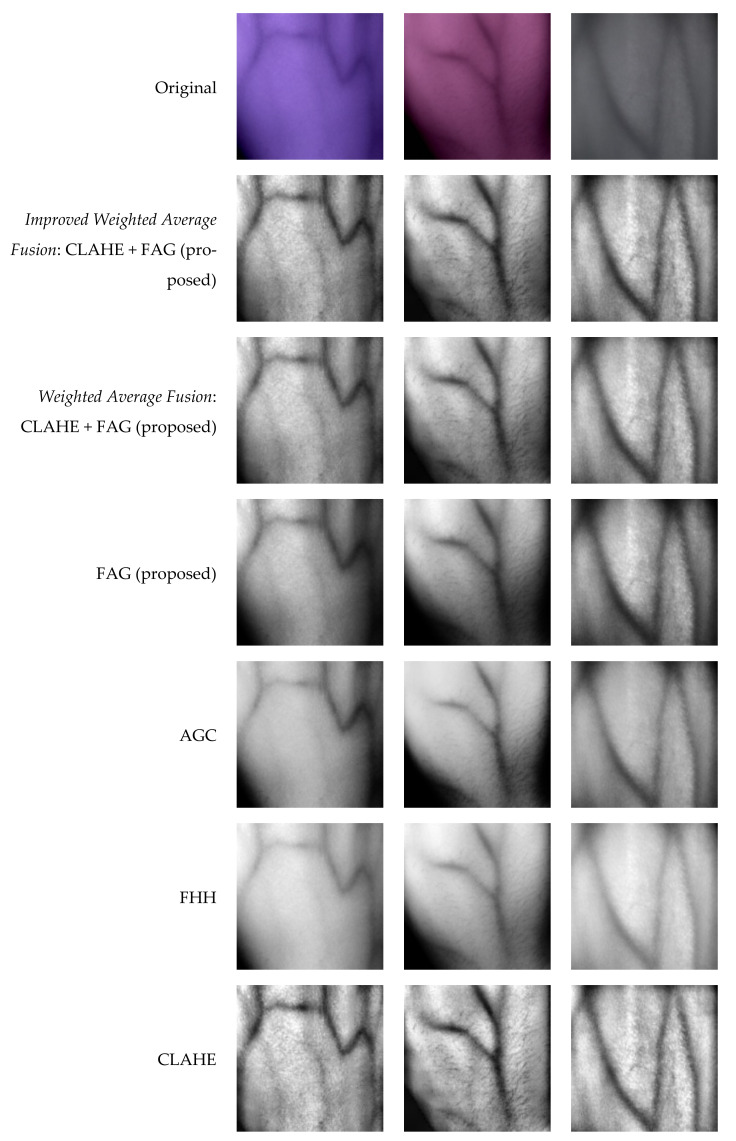
Sample results of various vein enhancement techniques based on three images from the SUAS database.

Figure 9 shows the enhanced images obtained by various image enhancement techniques on the SUAS database. CLAHE enhances pixel values and contrast of images but causes over-enhancement in smooth regions, particularly in the background. FHH and AGC have almost the same performance in visual quality, although they employ different mechanisms. In addition, FHH and AGC have produced a brighter image than the original input image. Although a combination of fuzzy and AGC in FAG yielded acceptable results, some vein edges remained blurred. The fusion of CLAHE + FAG with weighted average and the improved weighted average, on the other hand, produced comparable results. However, the fusion of CLAHE + FAG with improved weighted average produces a visually pleasing appearance after contrast enhancement by attaining less over-enhancing and blurriness than the fusion of CLAHE + FAG with standard weighted average.

Table 1 shows the average quantitative results of the six techniques in terms of MSE, PSNR, and SSIM for the two databases. The highest results of PSNR and SSIM and the lowest results of MSE are marked in bold in the table. It can easily be found that the proposed enhanced image has greater MSE and PSNR values for both databases than other techniques. This indicates that the proposed technique has better contrast.

### 4.3. Binary Analysis

The average performance measures of the proposed technique with a comparison of other enhancement techniques are listed in Table 2. Segmented hand vein patterns without any enhancement technique achieved 0.6035, 0.9272, and 0.6893 for Se, Acc, and Dice on the self-acquisition database. The proposed enhancement technique achieved 0.6880, 0.9422, and 0.7304 for Se, Acc, and Dice, respectively, outperforming all other techniques. In particular, with the proposed image enhancement, the detection of vein patterns improved 14 percent for Se, 1.6 percent for Acc, and 5.96 percent for Dice. Thus, the proposed enhancement technique has significantly enhanced the contrast and improved the detection of vein patterns.

Segmented hand vein patterns without any enhancement technique on the SUAS database achieved 0.5205, 0.9374, and 0.6334 for Se, Acc, and Dice. The proposed enhancement technique achieved 0.6783, 0.9559, and 0.6705 for Se, Acc, and Dice, respectively, with the results for Se and Acc being better than other techniques. Although the Dice is not the best result, the detection of vein patterns using the proposed enhancement technique improved 30 percent for Se, 1.97 percent for Acc, and 5.85 percent for Dice. Thus, the comparison results in Table 2 indicate that the proposed enhancement technique has significantly enhanced the contrast and improved vein patterns detection.

**Figure 10:**
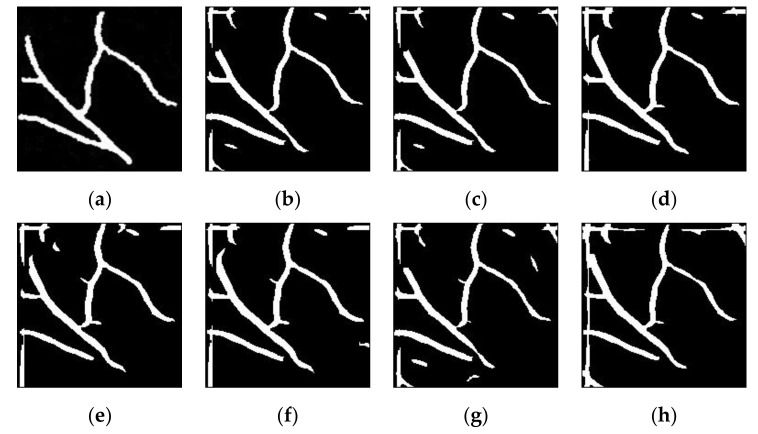
Sample results of vein detection for various image enhancement techniques on a self-acquisition database: (**a**) truth image; (**b**) improved weighted average fusion CLAHE + FAG(proposed); (**c**) weighted average fusion CLAHE + FAG(proposed); (**d**) FAG(proposed); (**e**) AGC; (**f**) FHH; (**g**) CLAHE; (**h**) without enhancement.

**Figure 11:**
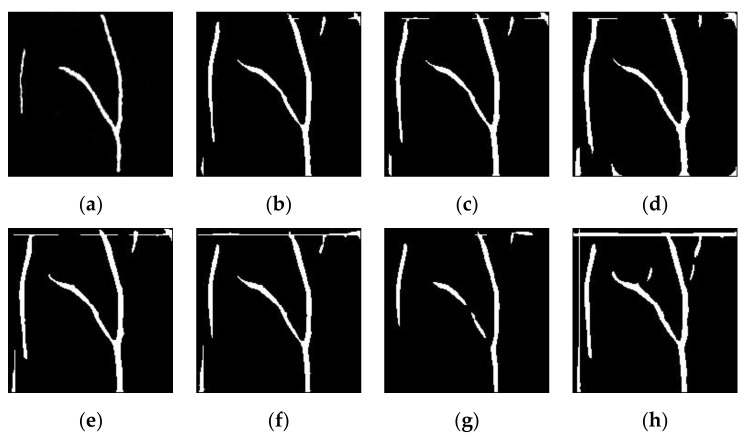
Sample results of vein detection for various image enhancement techniques on the SUAS database: (**a**) truth image; (**b**) improved weighted average fusion CLAHE + FAG(proposed); (**c**) weighted average fusion CLAHE + FAG(proposed); (**d**) FAG(proposed); (**e**) AGC; (**f**) FHH; (**g**) CLAHE; (**h**) without enhancement.

Sample results of vein segmentation on the self-acquisition database and SUAS database are shown in Figure 10 and Figure 11, respectively. The segmentation process on low contrast images (without any enhancement technique) could make it difficult for MF-FDOG to distinguish vein pixels and background pixels. As a result, more unwanted noise is generated in the segmented image. In contrast, it can be seen that the proposed technique in Figure 10b and Figure 11b has succeeded in identifying the vein patterns as it produces resultant images that are mostly similar to its corresponding ground-truth image for both databases.

## 5. Conclusions

In this paper, an improved weighted average fusion of CLAHE + FAG was proposed to improve NIR hand vein image enhancement. The proposed technique produces an enhanced vein image by making it into three steps. First, the CLAHE algorithm was used as the first technique to enhance the contrast between veins and the background. Second, a global technique of FAG in the fuzzy domain was proposed. FAG enhances the bright and dark of hand vein images using different transformation functions. Finally, by taking advantage of both spatial domain and fuzzy domain techniques to improve the enhancement of hand vein images, a fusion approach consisting of CLAHE and FAG using an improved weighted average was proposed. Experiments were conducted qualitatively and quantitatively in the enhancement and segmentation stages. The experimental result shows that the proposed enhancement technique can significantly enhance vein patterns and improve detection in vein pattern segmentation.

## Figures and Tables

**Table 1:** Average results in terms of MSE, PSNR, and SSIM for various image enhancement techniques. The best values are indicated in bold.

Enhancement Techniques	Self-Acquisition Database			SUAS Database		
	MSE	PSNR	SSIM	MSE	PSNR	SSIM
CLAHE	929.78	18.5316	0.7165	5025.30	11.2784	0.4596
FHH	2045.10	15.5779	0.8278	5674.44	11.2768	**0.8144**
AGC	6560.91	10.6032	0.7207	5748.90	10.6239	0.7769
FAG(proposed)	1287.09	17.7666	**0.8794**	5681.23	12.0139	0.7784
Weighted Average Fusion CLAHE + FAG(proposed)	797.28	19.6792	0.8012	3182.04	13.8934	0.6831
Improved Weighted Average Fusion CLAHE + FAG(proposed)	**682.24**	**20.2106**	0.8048	**3117.07**	**13.9779**	0.6072

**Table 2:** Average results of vein segmentation using various techniques of enhanced images on two databases. The best values are indicated in bold.

Enhancement Techniques	Self-Acquisition Database			SUAS Database		
	Se	Acc	Dice	Se	Acc	Dice
Without enhancement	0.6035	0.9272	0.6893	0.5205	0.9374	0.6334
CLAHE	0.6665	0.9385	0.7178	0.6552	0.9556	0.6676
FHH	0.6561	0.9375	0.7190	0.6015	0.9503	**0.6810**
AGC	0.6379	0.9323	0.6930	0.5700	0.9445	0.6401
FAG(proposed)	0.6729	0.9402	0.7262	0.6139	0.9492	0.6515
Weighted Average Fusion CLAHE + FAG(proposed)	0.6875	0.9418	0.7279	0.6705	0.9557	0.6800
Improved Weighted Average Fusion CLAHE + FAG(proposed)	**0.6880**	**0.9422**	**0.7304**	**0.6783**	**0.9559**	0.6705

## Data Availability

Not applicable.
